# Special Feature: Diversity of Insect-Plant Interactions in the Eastern Andes of Ecuador

**DOI:** 10.1673/031.009.2601

**Published:** 2009-06-02

**Authors:** James S. Miller, Lee Dyer

**Affiliations:** ^1^ Department of Biology, University of Nevada, Reno, NV 89557; ^2^ American Museum of Natural History, Division of Invertebrate Zoology, New York, NY

Over half the described organisms in the world are involved in plant-insect-parasitoid interactions (Hawkins and Sheehan 1994), and these provide the basis for our understanding of fundamental issues in ecology and evolutionary biology (e.g., Hairston et al. 1960; Ehrlich and Raven 1964; Janzen 1973; Lawton and McNeill 1979; Price et al. 1980; Karban and Myers 1989; Gentry and Dyer 2002). In fact, relationships between parasitoids, herbivorous insects, and their host plants are among the most productive systems for understanding multi-trophic interactions (e.g., Abrahamson and Weiss 1997; English-Loeb et al. 1993; Hochberg and Ives 2000; Jervis and Kidd 1996; Quicke 1997; Singer and Stireman 2003; Whitfield 1998, 2003). Documenting these ubiquitous systems has lagged far behind more traditional biotic surveys and inventories. The resulting lack of basic natural history data has led to unsubstantiated paradigms, as well to numerous disagreements in ecology and evolutionary biology. For example, it has been assumed commonly that herbivorous insects are highly host-specific (reviewed by Futuyma and Moreno 1988; Jaenike 1990; Irschick et al. 2005), especially in the tropics. More recently, however, this paradigm has been challenged by the results of an extensive survey of herbivore-plant interactions (Novotny et al. 2006), sparking a vigorous debate (Dyer et al. 2007; Stork 2007). Collecting and inventory methods that rely on rearing wild-caught specimens preserve much of the ecological, environmental and behavioral context of specimens, while also providing high quality taxonomic data.

*Caterpillars and Parasitoids of the Eastern Andes in Ecuador* is an ongoing, long-term rearing project dedicated to the inventory and dissemination of information on lepidopteran larvae, their host plants and their parasitoids. It provides important natural history data from a uniquely diverse and highly threatened ecosystem. This survey, officially begun in 2001 with funds provided by Earthwatch Institute, has continued with funding from a variety of sources, including National Geographic and the National Science Foundation. It is part of a coordinated transAmerican effort taking place at sites in Ecuador, Costa Rica, Arizona and Louisiana (Dyer et al. 2007; Stireman et al. 2005; Gentry and Dyer 2002; www.caterpillars.org). The primary goals of the project are:

- To survey and inventory a diverse community of Lepidoptera and their associated parasitoid Hymenoptera and Diptera, thus providing specimens for future morphological and molecular systematic research.
- To collect baseline natural history information documenting caterpillar-host plant-parasitoid relationships, larval development rates, and other life cycle information for described and undescribed species. These data can be used to test ecological and evolutionary hypotheses.
- To disseminate this information *via* a searchable database, publicly accessible through the internet and available worldwide.

Ecuador is at the edge of the biological frontier when it comes to our understanding of insect-plant biodiversity and the taxonomic composition of tropical biological communities. On the slopes of the equatorial Andes, considered by many to be home to the highest levels of species endemism in the world (e.g., Brehm et al. 2005), most plant and animal groups remain poorly studied (Suarez and Ulloa 1993; Kessler 2000; Cresswell et al. 1999). Due to rapid rates of habitat destruction and growing human populations, these montane habitats are under immediate danger of deforestation (Suarez and Ulloa 1993). Areas of relatively low relief, more suitable for cattle grazing, are under particular threat.

Yanayacu Biological Station (YBS) is located at 2200 meters in the Quijos Valley, Napo Province, in the Andes of northeastern Ecuador (00°35.9*′*S, 77°53.4′W). The intermediate elevation of the station (situated in montane wet forest *sensu* Holdridge et al. 1971) provides easy access to a unique diversity of habitats, ranging from paramo (3800 m) to lowland rain forest (800 m and below). Much of the 2000 hectares encompassed by the YBS comprises flat, pristine cloud forest, some of the last remaining habitat of this type found anywhere in Ecuador.

This series of articles showcases basic natural history information, as well as complex tri-trophic interactions, in a poorly known fauna (Neotropical caterpillars and host plants, as well as their parasitic Hymenoptera and Diptera). Integrated information of this sort, novel even for well-studied faunas, is crucial for those wanting to test hypotheses involving tropical taxa. In this special feature, adult Lepidoptera and parasitoids are associated with their immature stages, providing new taxonomic characters for the families Geometridae, Nymphalidae, Hesperiidae, Tachinidae and Braconidae, groups for which the systematics and biodiversity are poorly known (e.g., Scoble et al. 1995; Penz 1999; Dolphin and Quicke 2001; Whitfield et al. 2001; Penz and DeVries 2002; Greeney and Jones 2003). The information generated by this project has also allowed examination of diversity patterns at larger geographical scales (see Dyer et al. 2007; Connahs et al. this issue) and across habitat types, because the sampling protocol includes Andean habitats broadly distributed along a north-south axis and across an elevational gradient.

These papers are a first attempt to summarize some of the taxonomic and ecological results from the rearing inventory in Ecuador. The overall collecting effort encompasses a huge variety of caterpillar and host plant taxa, but certain focal groups were particularly well sampled. These include the host plant genera *Piper* (Piperaceae) and *Chusquea* (Poaceae), lepidopteran larvae in the genus *Eois* (Geometridae), as well as caterpillars in the butterfly families Hesperiidae and Nymphalidae. Future features will provide broad syntheses treating additional taxonomic groups, such as the Arctiidae, Saturniidae, Sphingidae, Noctuidae and Limacodidae, as the data accumulate and species identifications become further refined.
